# Zinc supplementation reduces *Candida* infections in pediatric intensive care unit: a randomized placebo-controlled clinical trial

**DOI:** 10.3164/jcbn.18-74

**Published:** 2018-11-30

**Authors:** Jun Xie, Lihong Zhu, Tingli Zhu, Ying Jian, Ye Ding, Min Zhou, Xiaoyan Feng

**Affiliations:** 1 Nursing Department, Wuxi Children’s Hospital, Wuxi 214023, P. R. China

**Keywords:** zinc supplementation, pediatric intensive care unit, candidemia, candiduria, *Candida* infection

## Abstract

Resistance to anti-fungal drugs has become the main cause for increasing incidence rate of *Candida* infections in pediatric intensive care units (PICU). Zinc supplementation has been shown to exhibit beneficial effects on many pediatric illnesses. This study aimed to investigate the efficacy of zinc supplementation on prevalence of candidemia and candiduria infections in PICU. 724 eligible children between 1 to 5 years old admitted in PICU were randomly assigned into either zinc supplementation group or placebo group. Primary endpoints were the number of *Candida* infections, length of PICU stay and cases of patient death 14 days after enrollment. Secondary endpoints were the incidence rates of candidemia and candiduria. The incidences of candiduria and candidemia were significantly lower in the zinc group than the placebo group. The length of PICU stay and cases of patient death were obviously lowered in the zinc group compared to the placebo group. In conclusion, zinc supplementation shows beneficial clinical efficacy in reducing *Candida* infections among PICU patients on broad-spectrum antibiotics with critical illnesses.

## Introduction

*Candida* infection in the bloodstream is a type of nosocomial infectious disease that has gained increasing research attention.^(1,2)^ Particularly in pediatric intensive care units (PICUs), the incidence rate of Candidemia has increased greatly in recent years.^(3,4)^ Data from several studies concluded that *Candida* species accounted for approximately 8–10% of bloodstream infections found in PICUs.^(5–7)^ According to the National Nosocomial Infections Surveillance System study, *Candida* species are responsible 85% of fungal infections among patients in critical conditions.^(8)^ Candidemia commonly results in high rate of morbidity and mortality, as well as high medical expense, among patients who are hospitalized.^(9)^ *Candida* colonization in the gastrointestinal tract has also been suggested to be correlated with elevated risk of invasive candidiasis.^(2,10,11)^ Keeping *Candida* colonization in check using systemic anti-fungal drugs has been successful in controlling the prevalence of invasive fungal infections.^(12–14)^ However, although anti-fungal treatment has exhibited potent clinical efficacy, it is not widely adopted as a routine clinical approach because of its adverse effects, tolerability and emergence of resistant strains that limit the effect of anti-fungal drugs.^(15,16)^ In this context, novel approaches need to be developed to supplement current anti-fungal treatment in order to reduce *Candida* colonization and subsequently prevent invasive candidiasis.

Zinc is a trace element required for normal function of many transcription related factors and enzymes, and is essential for all tissues and cells.^(17)^ It is reported that in pediatric critical illness, zinc homeostasis is disrupted,^(17)^ and zinc supplementation could protect against sepsis and infections among patients with compromised immune system,^(18)^ and significantly reduce mortality caused by infectious disease among pre-maturely newborn infants.^(19)^ Zinc deficiency was also commonly found in infants with severe pneumonia,^(20)^ while daily supplementation with 20 mg zinc in infants with severe pneumonia enhanced recovery, and reduced resistance to anti-microbials through decreasing exposure to broad-spectrum anti-biotics.^(21)^ Similarly, in studies among children with diarrhoea, zinc therapy reduced stool output, duration of diarrhea and reduced the use of antibiotics.^(22,23)^ In addition, in pediatric patients with shigellosis, a 14 day course of zinc supplementation improved inflammatory responses.^(24)^

However, although the beneficial effects of zinc supplementation have been widely suggested in many pediatric diseases, no investigation has been conducted to address its clinical efficacy in children admitted in PICU. The current randomized placebo-controlled clinical trial is designed to study the clinical effect of zinc supplementation on infections of candidemia and candiduria in PICU.

## Methods

### Ethical statements

This clinical trial was designed conforming with the guidelines stated in the Declaration of Helsinki, and was approved by the Ethical Committee of Wuxi Children’s Hospital. Written informed consent forms were acquired from parents/guardians of all patients, who also agreed to the policy of anonymous data utilization.

### Patients

Between April and October each year from 2013 to 2017, a total of 857 children between 1 to 5 years old, who were admitted into the PICU of Wuxi Children’s Hospital and prescribed with broad-spectrum antibiotics for over 2 days, were enrolled. 133 patients with history of anti-fungal treatments immunodeficiency, known chronic illnesses, known gastrointestinal diseases or previous zinc supplements were excluded.

### Randomization and intervention

724 eligible patients remained in the trial after exclusion, and they were randomly assigned into either zinc group (*n* = 358) or placebo group (*n* = 366), according to a permuted-block randomizing algorithm stratified to their age at admission. Patients were administered with either 20 mg elemental zinc per day (10 mg zinc per 5 ml syrup) or placebo syrup, respectively, for 14 days. Containers of zinc-supplemented and placebo syrups were identical make their contents blind to both the investigators and patients.

### Anti-fungal treatment

Clinically stable candiduria patients were prescribed with fluconazole, while candidemia patients were prescribed with IV amphotericin B. Patients showing severe sepsis signs after at least 5 days of broad-spectrum antibiotics treatment were prescribed with amphotericin B before fungal culture reports were obtained.

### Definition of endpoints

All assessments of outcomes were conducted at admission of PICU and 14 days after the start of zinc supplementation, by investigators blind to the group assignment. In clinically indicated infection cases, blood samples were collected by venipuncture for fungal and bacterial culture, while urine samples were collected in sterile urine bags or through urinary catheter for fungal and bacterial culture. Blood zinc concentration was quantified using inductively coupled plasma mass spectrometry (Thermo Fisher, Waltham, MA). For fungal culturing, blood sample was transferred into two culturing bottles containing Sabouraud agar with mg/L gentamicin 20 and 50 mg/L chloramphenicol, and incubated first at 22°C for 7 days and then at 37°C for another 7 days. Growth of fungal culture was measured by urease test, germ tube test, sporulation on corn meal agar, sugar assimilation and sugar fermentation tests. Candidemia was defined as *Candida* species isolated from blood sample cultures, while candiduria was defined as *Candida* species isolated from urine sample cultures. Primary endpoint was the incidence rate of *Candida* colonization (*Candida* isolated from rectal swab) 14 days after trial initiation. Secondary endpoint was *Candida* growth in blood (candidemia) or urine (candiduria) samples.

### Statistical analysis

Statistical analysis was conducted using SPSS software package (SPSS, Chicago, IL). Descriptive statistical analysis, including mean, standard deviation, median, range and percentages, were employed to quantify baseline variables. Continuous variables were analyzed by two tailed student *t* test. Statistical differences between the two groups were analyzed by either chi-square test or Mann-Whitney test if applicable. Number of patients recruited was determined using established statistical power analysis.^(25)^ Briefly, differences between means of each compared data set were divided by their standard deviation, generating the standardized effect size. Next, the minimum required sample size was calculated using 5% as significance level and 90% power. *P*<0.05 indicates statistically significant difference.

## Results

**Fig. 1:**
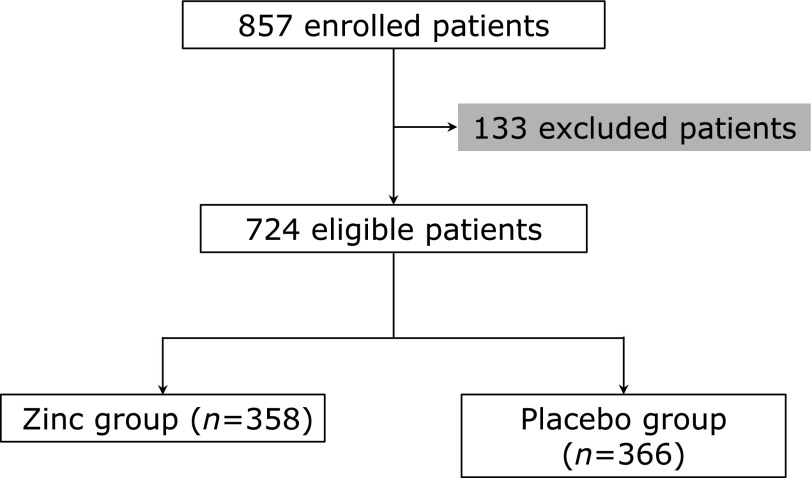
Illustration of study design.

Figure 1 illustrated the flowchart of the study. At first, a total of 857 patients were recruited, among which 133 patients were excluded. The 724 eligible patients remained in the study were assigned into either zinc group (*n* = 358) or placebo group (*n* = 366). Table 1 listed clinical and demographic characteristics, as well as intervention methods used in both treatment groups. There were no statistical differences between the two groups of patients at PICU admission, in terms of demographic features (age, gender and body weight), blood zinc concentration and clinical manifestations. Importantly, in analyzing the risk factors of invasive fungal infections, such as illness severity, invasive catheters, mechanical ventilation and urinary catheterization, as well as *Candida* infection status, were also found to be indistinguishable between the two groups (Table 1).

As shown in Table 2, zinc supplementation significantly increased the blood zinc concentration of zinc group patients, compared to that of placebo group. Importantly, zinc supplementation also reduced the prevalence of candidemia, as indicated by 10 patients in the zinc group with candidemia compared to 22 found in the placebo group (*p* = 0.03). Candiduria prevalence in the zinc group (*n* = 37) was also markedly lower than that of the placebo group (*n* = 91) (*p* = 0.02). Next, the numbers of patients from the zinc group with nosocomial urinary tract infection (*n* = 57) and bloodstream infection (*n* = 64) were significantly reduced compared with those of the placebo group (*n* = 91 *p* = 0.01 and *n* = 94 *p* = 0.03, respectively). 41 patients in the zinc group, while 85 patients in the placebo group, received anti-fungal therapy, which was significantly higher (*p* = 0.03). The days of patients on treatment of broad-spectrum antibiotics in the zinc group was also greatly reduced compared to the placebo group (6.2 ± 2.3 days vs 10.2 ± 3.1 days, *p* = 0.02). The length of PICU stay (10.7 ± 2.5 days) and cases of patient death (*n* = 17) were also obviously lowered in the zinc group compared to the placebo group (16.1 ± 3.2 days, *p* = 0.03; *n* = 29, *p* = 0.02), which was in agreement with earlier report where zinc supplementation significantly reduced mortality from infectious disease among infants.^(19)^

The pathogenic candidemia species in the blood were also analyzed (Table 3). 6 cases of *Candida albicans*, 3 cases of *Candida tropicalis* and 1 case of *Candida guilliermondii* were identified in the zinc group. On the other hand, 14 cases of *Candida albicans*, 5 cases of *Candida tropicalis* and 3 case of *Candida guilliermondii* were identified in the placebo group, all markedly higher than the zinc group. Similarly, among candidemia species in the urine (Table 4), numbers of infections isolated were all significantly reduced in the zinc group compared to those in the placebo group.

## Discussion

This trial was designed to investigate the potential efficacy of zinc supplementation against *Candida* infections in PICU patients who were critically ill and on broad-spectrum antibiotic treatments. Data from the trial suggested that, a 14 day course of zinc supplementation significantly reduced incidence rate of both candiduria and candidemia, compared to placebo. We therefore could conclude that oral zinc supplementation is able to control invasive candidiasis in critically ill PICU patients.

Results obtained from prophylactic use of systemic anti-fungal drugs have indicated that restricting colonization of *Candida* could contribute to the prevention of invasive candidiasis.^(15,16)^ It is reported that in high-risk preterm neonates, a miconazole oral gel could reduce gut fungal colonization by over 60%,^(14)^ while intravenous fluconazole could reduce rectal colonization by ~75%.^(13)^ However, concerns on the preventive utilization of anti-fungal agents, such as side effects, selection of resistant strains, and elevated medical expenses, have restricted their clinical use.^(15)^ In line with this, novel alternative or adjuvant therapies are urgently needed to facilitate the combat against *Candida* colonization.

The role of zinc has been implicated in severe pediatric diseases, where plasma zinc concentrations were found low in critically ill children.^(17)^ *Candida* species are a type of commensal micro-organisms, which constitute for approximately 20–50% of all microorganisms in the oral cavities of a healthy individual.^(26)^ Among all types of human candidiasis, *Candida albicans* is deemed as the species with the highest pathogenicity.^(27)^ On the other hand, *Candida* spp. was reported to be the most common fungal species accountable for blood stream infections of the central line.^(28)^ Although amphotericin B and fluconazole were largely effective against *Candida albicans* and *Candida tropicalis*, therapy failure and resistance to fluconazole have been reported.^(29)^ Emergence of resistant *Candida* strains therefore requires new anti-fungal therapies.^(30)^ Interestingly, zinc limitation has recently been reported to induce a hyper-adherent phenotype in *Candida albicans*, suggesting zinc might play a positive role in antagonizing *Candida albicans* pathogenicity.^(31)^ Importantly, zinc oxide nanoparticles exhibited anti-microbial efficacy against microorganisms including *Candida albicans*.^(32)^

Among all findings of the current trial, the most important result is that, zinc supplementation has decreased candiduria prevalence by nearly 50%. Since candiduria is a key biomarker for severe colonization and systemic candidiasis,^(33,34)^ especially among critically ill children,^(35)^ our observation on reduced candiduria prevalence therefore strongly demonstrated potent protective effect of routine zinc supplementation against invasive infections of candidiasis. In addition, the double-blind, randomized, and placebo-controlled study design, together with a reasonable large sample pool, has brought statistical accuracy and confidence in the reliability of our data.

## Conclusion

We hereby report that, for the first instance, zinc supplementation exhibits potent beneficial effect in reducing *Candida* infections among critically ill PICU patients receiving broad-spectrum antibiotics. The clinical efficacy of adjuvant prescription of zinc and antibiotics is therefore supported by our data, which is potentially able to serve as a novel therapeutic approach to control *Candida* infections in PICU.

## Figures and Tables

**Table 1:** Baseline demographics and clinical characteristics of patients at PICU admission

Characteristics	Zinc (*n* = 358)	Placebo (*n* = 366)	*p* value
Age (years, mean ± SD)	4.3 ± 0.3	4.1 ± 0.4	0.31
Gender (boy/girl)	182/176	188/178	0.43
Body weight (kg, mean ± SD)	15.2 ± 1.9	14.3 ± 2.4	0.28
Blood zinc concentration (mg/L, mean ± SD)	6.9 ± 1.8	7.0 ± 1.9	0.34
Central nervous system infections (*n*)	191	198	0.51
Community-acquired pneumonia (*n*)	112	107	0.26
Cardiac diseases (*n*)	29	26	0.35
Others (*n*)	26	22	0.2
Pediatric risk of mortality III, median (10–90th centile)	13 (3–20)	12 (2–21)	0.54
Glasgow Coma Scale, median (10–90th centile)	7 (4–11)	7 (4–10)	0.37
Mechanical ventilation (*n*)	316	302	0.17
Central catheter (*n*)	328	311	0.29
Urinary catheter (*n*)	331	324	0.34
Candidemia (*n*)	43	41	0.51
Candiduria (*n*)	71	67	0.59

**Table 2:** Patient outcomes

Outcomes	Zinc (*n* = 358)	Placebo (*n* = 366)	*p* value
Blood zinc concentration (mg/L, mean ± SD)	9.2 ± 2.6	7.3 ± 2.0	0.02
Candidemia (*n*)	10	22	0.03
Candiduria (*n*)	37	80	0.02
Nosocomial urinary tract infection (*n*)	57	91	0.01
Nosocomial bloodstream infection (*n*)	64	94	0.03
Treated with anti-fungal drug (*n*)	41	85	0.03
Broad-spectrum antibiotics treatment (days, mean ± SD)	6.2 ± 2.3	10.2 ± 3.1	0.02
PICU stay (days, mean ± SD)	10.7 ± 2.5	16.1 ± 3.2	0.04
Death (*n*)	17	29	0.02

**Table 3:** *Candida* isolated from blood

Outcomes	Zinc (*n* = 358)	Placebo (*n* = 366)
*Candida albicans* (*n*)	6	14
*Candida tropicalis* (*n*)	3	5
*Candida guilliermondii* (*n*)	1	3

**Table 4:** *Candida* isolated from urine

Outcomes	Zinc (*n* = 358)	Placebo (*n* = 366)
*Candida albicans* (*n*)	23	54
*Candida tropicalis* (*n*)	9	15
*Candida guilliermondii* (*n*)	5	11
